# Favourable response of Blaschko linear psoriasis to interleukin-23 inhibition

**DOI:** 10.1093/skinhd/vzae010

**Published:** 2025-01-22

**Authors:** Jeremias L K Reich, Alexandros Onoufriadis, John A McGrath, Kristian Reich

**Affiliations:** Oxford University Clinical Academic Graduate School, Oxford University, Oxford, UK; Laboratory of Medical Biology and Genetics, School of Medicine, Aristotle University of Thessaloniki, Thessaloniki, Greece; School of Basic and Medical Biosciences, St John’s Institute of Dermatology, King’s College London, London, UK; Center for Translational Research in Inflammatory Skin Diseases, Institute for Health Services Research in Dermatology and Nursing, University Medical Center Hamburg-Eppendorf, Hamburg, Germany

Dear Editor, More than 60 genomic loci have been described at which variation modulates susceptibility to plaque-type psoriasis in European populations.^1^ The primary risk allele is HLA-C*06:02 which may be involved in the presentation of autoantigens.^2,3^ Some therapies for psoriasis specifically target mediators such as interleukin (IL)-12 and/or IL-23, which exert possible educational functions during antigen presentation, and genetic factors associated with psoriasis may also be useful in the identification of responders to anticytokine therapies. In fact, individuals carrying the HLA-C*06:02 allele are more likely to respond to the IL-12/23p40-inhibiting antibody ustekinumab, less likely to respond to the anti-tumour necrosis factor (TNF) antibody adalimumab, and have little or no difference in response to the IL-17A-blocking antibody secukinumab.^4–6^

Psoriasis very rarely manifests along the lines of Blaschko, termed linear or Blaschko linear psoriasis (BLP; type I if appearing as the only manifestation of psoriasis; type II if overlapping with milder, widespread lesions of psoriasis vulgaris). BLP represents a genetic mosaicism in which lesional but not nonlesional skin contains the variation responsible for the manifestation of the phenotype. A potential mechanism is somatic genetic mosaicism, i.e. a *de novo* mutation in an embryonic epithelial cell and the cells that subsequently derive from it. BLP may therefore be viewed as a model for psoriasis with prominent contribution of genetic factors.^7^

We previously presented the transcriptomic analysis of skin biopsies of two patients with BLP that revealed large overlaps with psoriasis vulgaris and also suggested some potential differentiators.^8^ Here we report the clinical cases of these two patients, both negative for HLA-C*06:02, and their response to anti-IL-23p19 therapy, suggesting activation of the IL-23/IL-17 pathway in BLP.

The first patient is a 34-year-old man, diagnosed with type I BLP 10 years ago, who presented with linear erythematous plaques with thick scales in a lateralized Blaschkoid pattern, affecting the right trunk and arm (Figure 1a). Biopsies taken from untreated lesional skin confirmed psoriasis. The patient had tried several cycles of therapy with topical corticosteroids and calcipotriol, as well as bath psoralen + ultraviolet A without improvement. He was started on the IL-23p19 inhibitor risankizumab. There was slow but significant improvement over the first year of treatment; Psoriasis Area and Severity Index (PASI) decreased from 24.6 at baseline to 2.1 after 56 weeks of therapy (Figure 1b).

**Figure 1:**
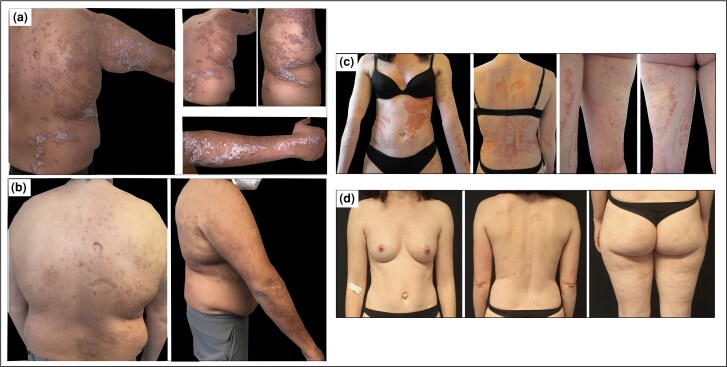
Clinical images of patient 1 at (a) baseline and (b) 56 weeks of treatment with risankizumab; and patient 2 at (c) baseline and (d) after 4 months of treatment with guselkumab.

The second patient is a 38-year-old woman with type I BLP since early childhood. She had tried topical corticosteroids/calcipotriol and ultraviolet B narrow-band phototherapy with limited benefit. Systemic treatment with fumaric acid esters initially improved the disease, but disease control was lost after several years and stopped when the patient planned to conceive. Leaving topical corticosteroids as the only therapy, the psoriasis remained poorly controlled. Physical examination revealed erythematous, scaly plaques displaying a chequerboard pattern on the left abdomen, and a linear narrow band Blaschkoid pattern on the right abdomen, back and the extremities (Figure 1c). Skin biopsies taken from untreated lesional skin showed typical features of psoriasis. After her pregnancy, the patient was started on the IL-23p19 inhibitor guselkumab. PASI improved from 20.5 at baseline to 2.6 after 4 months (Figure 1d), and the histology normalized.

BLP is very rare and only a few cases have been published.^9^ Histological features of BLP are identical to those of classical plaque-type psoriasis and help to distinguish it from important differentials, such as inflammatory linear verrucous epidermal naevus (ILVEN).^9^ Little is known about the response of BLP to treatments used for classical psoriasis. Most case reports suggest an insufficient response to topical treatment and phototherapy and conventional anti­psoriatic agents, and there is also evidence for recalcitrancy to biologics, such as TNF-α inhibitors and ustekinumab.^9,10^

We report the first two patients with BLP successfully treated with IL-23p19 inhibitors; both patients achieved clear or almost clear skin. In line with the transcriptomic analysis of skin biopsies of these patients,^8^ and although interindividual differences are likely, these findings suggest that the IL-23/IL-17 pathway is active in BLP, which is interesting considering the assumed genetic component of BLP (somatic mutations, however, were not analysed in these two cases). IL-23, but not IL-12, is upregulated in psoriatic lesions^11^ and most likely produced by inflammatory monocyte-like cells.^12^ IL-23 release during interaction of antigen presenting cells (APCs) with T cell contributes to enhanced IL-17 production. The favourable clinical response of HLA-C*06:02 carriers to ustekinumab may be interpreted as related to a prominent role of antigen presentation in the disease process in these patients and a role of IL-23 during APC-driven T-cell activation. While data on HLA-C*06:02 carriage and response to IL-23p19 inhibitors is not available, the fact that our two BLP cases were negative for HLA-C*06:02 may indicate that the IL-23 pathway is active in psoriasis irrespective of HLA-C*06:02 status.

## Data Availability

The data underlying this article will be shared on reasonable request to the corresponding author.
